# The prognostic role of circulating tumor cells in gastric cancer: A meta-analysis

**DOI:** 10.3389/fonc.2022.963091

**Published:** 2022-10-13

**Authors:** Zuxi Li, Meijuan Song, Shangjun Han, Chuanwei Jin, Jing Yang

**Affiliations:** ^1^ The First Clinical School of Gansu University of Chinese Medicine, Lanzhou, China; ^2^ General Surgery Clinical Medical Center, Gansu Provincial Hospital, Lanzhou, China; ^3^ Gansu Provincial Key Laboratory of Molecular Diagnosis and Precision Therapy of Surgical Tumors, Gansu Provincial Hospital, Lanzhou, China

**Keywords:** gastric cancer, circulating tumor cell, CTC, prognosis, meta-analysis

## Abstract

**Objective:**

We conducted a meta-analysis to evaluate the relationship between circulating tumor cells (CTC) and the prognosis of patients with gastric cancer.

**Materials and methods:**

The cohort studies reporting on the relationship between CTC and prognosis of gastric cancer were collected from Pubmed, Cochrane, Embase, CNKI, WanFang Data, and VIP databases. The two researchers independently screened the literature, extracted the data, and evaluated the bias risk of the included literature. The data were analyzed by Revman software (Review Manager version 5.4).

**Result:**

A total of 14 retrospective cohort studies with 1053 patients were included. The results showed that the overall survival time (OS) and progression-free survival time (PFS) of CTC-positive patients were shorter compared to CTC-negative patients. Taking into consideration the critical value of CTC positive patients, country of origin, sample size, treatment mode, and study time, the subgroup analysis showed that CTC-positive was related to the shortening of OS in patients with gastric cancer. Based on the subgroup analysis of the factors such as CTC positive critical value < 2.8, sample size ≥ 75, mixed therapy, longer study duration, country, and immunofluorescence detection of CTC, it was found that OS in CTC positive group was shorter than that in CTC-negative group (all P<0.05), while the critical value of positive CTC ≥ 2.8, sample size ≥ 75, choice of treatment only for operation or non-operation, short study time and molecular detection of CTC were not associated with OS (all P>0.05). In addition, CTC-positive patients had a more advanced TNM staging, poorer tumor differentiation, and earlier distant metastasis.

**Conclusion:**

CTC can be used as a prognostic indicator of gastric cancer. Gastric cancer patients with positive CTC may have a poorer prognosis compared to those with CTC-negative tumors.

**Systematic Review Registration:**

https://www.crd.york.ac.uk/PROSPERO/, identifier CRD42022323155.

## Introduction

Gastric cancer is one of the most common malignant tumors (1) and the second deadliest tumor worldwide (2). Smoking tobacco, age over 60, *Helicobacter pylori* infection, alcohol consumption, and obesity are the main causes leading to a gastric tumor (3). Surgery is the most effective treatment, yet patients present with an advanced stage at the time of diagnosis, losing their chance to undergo surgical resection (4). Chemotherapy and immunotherapy are the most common treatment methods for advanced-stage gastric tumors (5). Still, most gastric patients develop metastasis after therapy and have a poor prognosis.

In recent years, with the development of liquid biopsy technology, several new biomarkers have been discovered for accurately predicting the prognosis of gastric cancer and effectively evaluating the efficacy of chemotherapy for gastric cancer. For example, circulating tumor cells (CTC), i.e., tumor cells that detach from the primary or metastatic focus of the tumor and enter the blood, have recently attracted interest as biomarkers of cancer metastases (6–9). At present, existing studies have shown that CTC has an important role in the diagnosis of early gastric cancer (10), the guidance of chemotherapy, and analysis of chemotherapy efficacy (11–15), chemotherapy resistance (16), and prognosis (17, 18). Clinically, CTC has incomparable potential value in evaluating the prognosis of tumors. Given the important value of CTC in evaluating the prognosis of malignant tumors, we performed a meta-analysis in order to determine the relationship between baseline CTC and the prognosis of patients with gastric cancer and objectively evaluate its prognostic value in gastric cancer.

## Materials and methods

### Retrieval strategy

Six electronic databases were explored: Pubmed, Cochrane, Embase, CNKI, WanFang Data, and VIP. The cohort studies reporting on the relationship between CTC and prognosis of gastric cancer were collected from the establishment of the database to December 26, 2021. The following key words were used (Pubmed database): Neoplasm Circulating Cells, Neoplasm Circulating Cell, Circulating Neoplastic Cells, Circulating Neoplastic Cell, Circulating Tumor Cells, Circulating Tumor Cell, Embolic Tumor Cell, Embolic Tumor Cells, Tumor Embolism, Tmor Embolisms, CTC, Stomach Neoplasms, Stomach Neoplasm, Gastric Neoplasms, Gastric Neoplasm, Cancer of Stomach, Stomach Cancers, Gastric Cancer, Gastric Cancers, Stomach Cancer, Cancer of the Stomach, Prognosis, Prognoses, Prognostic Factors, Prognostic Factor. Chinese keywords include: circulating tumor cells, CTC, gastric cancer, and prognosis. This study has been registered on PROSPERO platform (Registration number: CRD42022323155).

### Inclusion and exclusion criteria

Inclusion criteria for research literature were (1): studies evaluating the relationship between CTC expression and prognosis of gastric cancer (2); dividing patients into high expression group and low expression group of CTC (3); describing effective prognostic indicators (OS, DFS, RFS, and PFS) or related clinicopathological parameters (tumor size, differentiation, depth of infiltration, lymph node metastasis, distant metastasis, and tumor stage) (4); enough data to calculate the hazard ratio (HR) or odds ratio (OR) and 95% confidence interval (95%CI) (5); patients did not receive any treatment at baseline (6); blood was collected and CTC were tested before treatment.

Exclusion criteria were (1): case reports, conference summaries, reviews, editorials, and non-human studies (2); repeated publication (3); lack of HR or Tumor and its 95%CI, or unable to estimate these parameters.

### Literature screening and data extraction

Two researchers (ZL and MS) independently reviewed and analyzed the title and abstract of the study, screened the search results, and evaluated the full text of the research literature that met the inclusion criteria. All differences were resolved through group discussion or by inviting a third researcher (JY). Two researchers (ZL and MS) independently extracted the following data from each study: title, first author, year of publication, study time, country, sample size, sex, treatment, follow-up time, CTC positive threshold, outcome indicators, and outcome measurements.

### Evaluation of research quality

The included study was independently assessed for bias risk according to the Newcastle-Ottawa quantity (NOS). A study with a score of 6 or more was defined as a high-quality study (19).

### Statistical analysis

Revman software (Review Manager version 5.4) was used for statistical analysis, and Stata software (Stata12.0 version) was used for sensitivity analysis and publication bias. In order to evaluate the effect of CTC on the prognosis of gastric cancer, the standard errors of risk ratio (HR), OS, or PFS were extracted from the included literature. HR > 1 indicates that the prognosis of the positive group is worse than that of the negative group. The inverse variance method was used to combine HRs in the Revman software. Considering the heterogeneity between studies, the literature heterogeneity was judged by *I^2^* statistics and the Q test. When *P* < 0.1 or *I^2^* > 50%, the heterogeneity was significant, and the random effect model was used for meta-analysis; on the contrary, the fixed effect model was used for meta-analysis (20). Publication bias was tested by the Beg method and the Egger method (test level α = 0.05) (21).

## Results

### Data screening process and results

**Figure 1:**
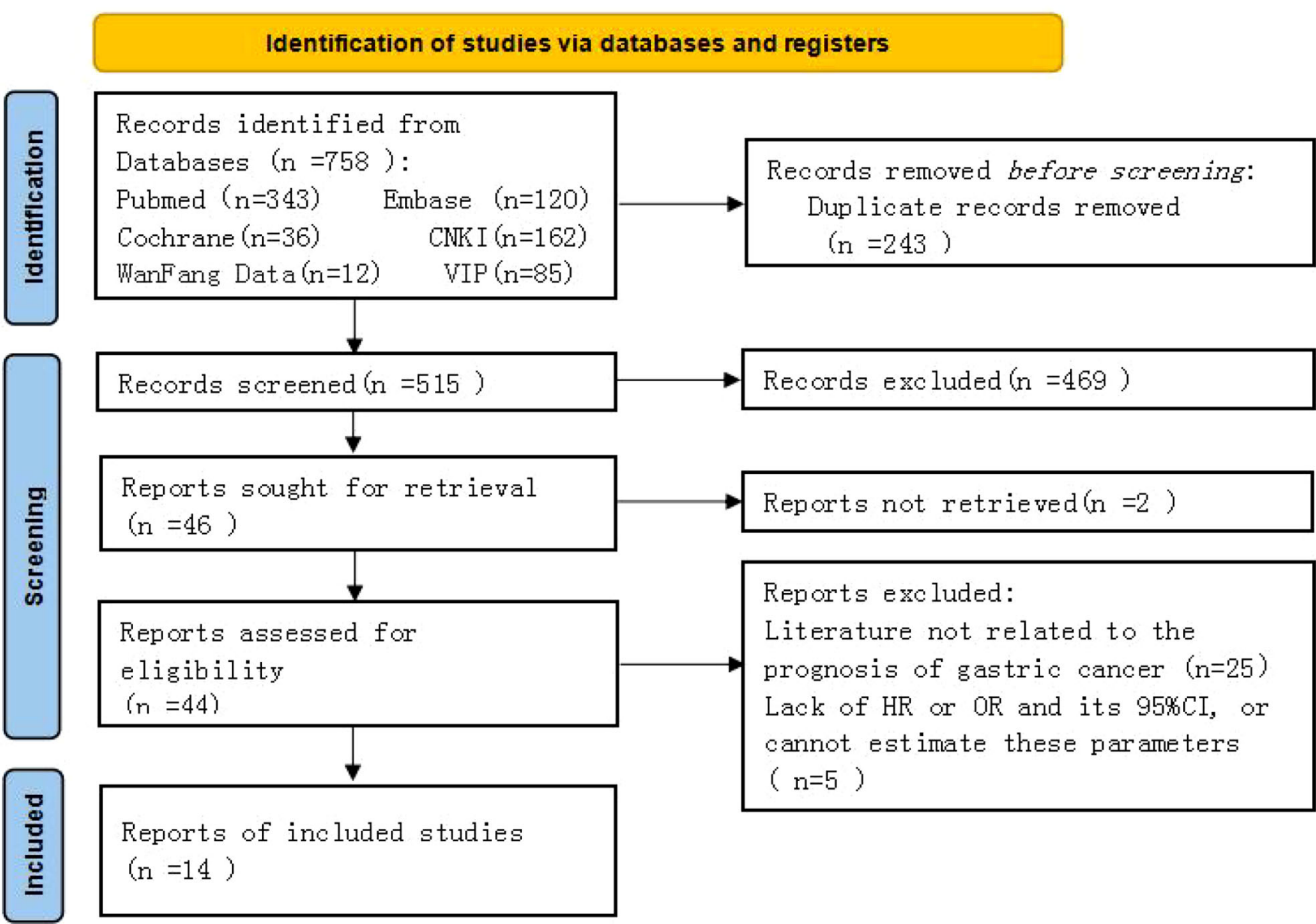
Literature screening process and results.

A total of 758 original studies were retrieved in the preliminary screening, 515 articles were obtained after deduplication, those that did not meet the inclusion criteria were excluded, and the full text was excluded after reading and evaluation. Finally, 14 retrospective cohort studies were included in the analysis (**Figure 1**).

### Basic characteristics of the included study

A total of 14 retrospective cohort studies were included (22–35), including 1053 patients. These studies were published from 2007 to 2021 (3 articles (23, 29, 35) were published in 2021). Six studies (23, 25, 26, 32, 34, 35) were conducted in China; others were carried out in Poland (22), Brazil (24), Germany (28), South Korea (29), Japan (27, 30, 31, 33) and other countries. Ten studies (22, 23, 26, 27, 29, 30, 32–35) reported the relationship between CTC and OS in gastric cancer patients, 3 (25, 28, 31) reported the relationship between CTC and OS or PFS in patients with gastric cancer, and 1 study (24) reported the relationship between CTC and PFS in patients with gastric cancer. The critical value of CTC positive was between 1 and 7.5. The NOS scores of the included studies were all above 6, indicating that the quality of the included studies was high (**Table 1**).

**Table 1:** Basic characteristics of the included study.

Included in the study	Research time(year)	Country	Sample size (n)	Male/female(n)	Mode of treatment	Follow-up time (months)	CTC detection method	Critical value of CTC positive	Outcome index	TNM staging	NOS score
Anna Pituch-Noworolska 2007	1997-1999	Poland	57	44/13	Operation	60	Immunofluorescence	3	OS	I-IV	8
Chengcheng Qian 2021	2016-2020	China	72	49/23	Mixed therapy	50	Immunofluorescence	1	OS	I-IV	8
Emne A.Abdallah 2019	2016-2017	Brazil	55	33/22	Mixed therapy	15	Immunofluorescence	2.8	PFS	I-IV	7
Huang Wei2019	2016-2017	China	28	16/12	Non- Operation	11	Immunofluorescence	4	OS、PFS	III-IV	7
Han Hongbing2015	2011-2013	China	60	36/24	Operation	18	Immunofluorescence	1	OS	I-IV	8
Hiroaki Ito 2016	2010-2011	Japan	65	46/19	Operation	60	Immunofluorescence	5	OS	I-IV	8
Ilja Kubisch 2015	2010-2011	Germany	62	39/23	Non- Operation	17	Molecular detection	1	OS、PFS	−	8
Joon Hyung Jhi 2021	2017-2018	South Korea	31	22/9	Non- Operation	12	Immunofluorescence	7.5	OS	−	7
Kunihiko Hiraiwa 2008	−	Japan	27	−	Mixed therapy	−	Immunofluorescence	2	OS	I-III	7
Okabe, H 2015	2008-2013	Japan	136	87/49	Mixed therapy	26	Immunofluorescence	1	OS、PFS	I-III	8
Qiyue Zhang 2018	2013-2014	China	93	68/25	Operation	36	Immunofluorescence	5	OS	I-III	8
Yoshikazu Uenosono 2013	2005-2012	Japan	148	99/49	Operation	60	Immunofluorescence	1	OS	I-IV	8
Yang Han2020	2014-2017	China	103	74/29	Mixed therapy	16.3	Immunofluorescence	2	OS	I-IV	7
Yinxing Zhu 2021	2015-2018	China	116	89/27	Mixed therapy	14.5	Molecular detection	3	OS	I-IV	7

### Meta-analysis results

#### Relationship between CTC and OS in patients with gastric cancer

**Figure 2:**
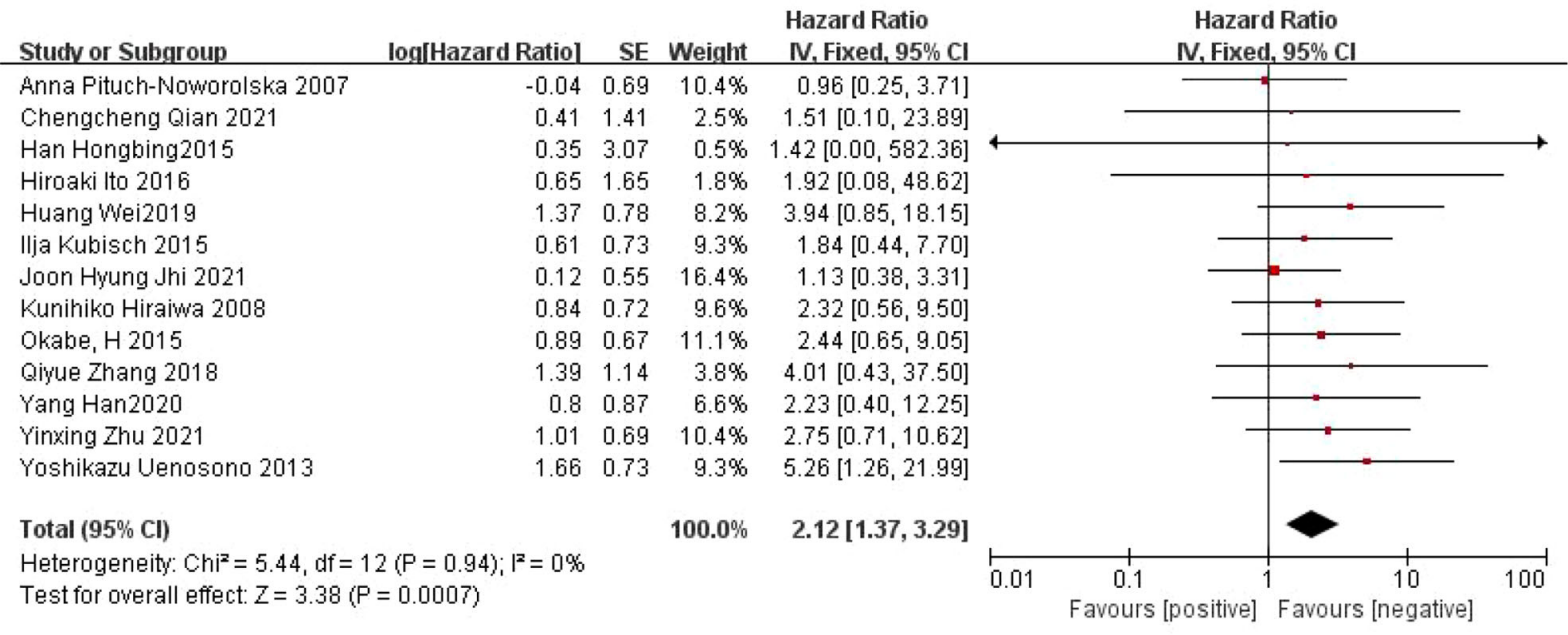
Meta-analysis of the relationship between CTC and OS.

A total of 13 articles (22, 23, 25–35) reported on the relationship between CTC and OS in patients with gastric cancer. There was no heterogeneity between the studies (*I^2 =^* 0%, *P*=0.94), and a fixed effect model was used. Meta-analysis showed that the OS was shorter in CTC-positive patients than in CTC-negative patients (*HR*=2.12, 95% *CI*=[1.37, 3.29], *P*=0.0007) (**Figure 2**).

#### Relationship between CTC and PFS in patients with gastric cancer

**Figure 3:**
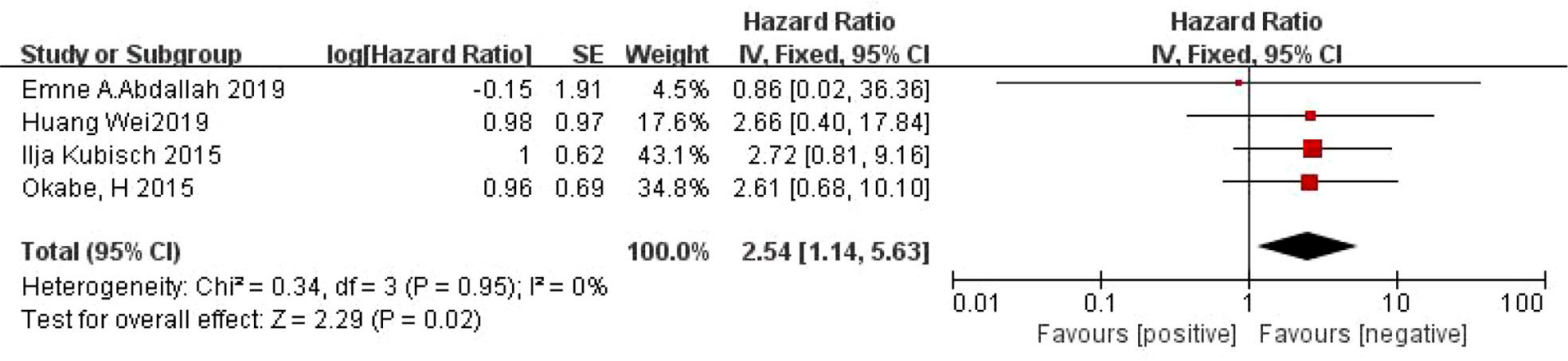
Meta-analysis of the relationship between CTC and PFS.

A total of 4 articles (24, 25, 28, 31) reported on the relationship between CTC and PFS in patients with gastric cancer. There was no heterogeneity between the studies (*I^2 =^* 0%, *P*=0.95), and a fixed effect model was used. Meta-analysis showed that the PFS was shorter in CTC-positive patients than in CTC-negative patients *(HR*=2.54, 95% *CI*=[1.14, 5.63], *P*=0.02) (**Figure 3**).

#### Subgroup analysis

In order to further analyze the prognostic effect of CTC on patients with gastric cancer, this study conducted a subgroup analysis taking into consideration the critical value, country, sample size, treatment mode, and research time of CTC positive. Subgroup analysis showed that when the critical value of CTC positive was < 2.8, the OS of the CTC positive group was shorter than that of a CTC-negative group; when the critical value of CTC was ≥ 2.8, there was no significant relationship between CTC and OS (all P<0.05). In addition, when the sample size was ≥ 75, the OS of the CTC-positive group was shorter than that of the CTC-negative group (P<0.05); when the sample size was < 75, there was no significant relationship between CTC and OS (P>0.05).

When the gastric cancer patients were treated with mixed therapy, the OS of the CTC-positive group was shorter than that of the CTC-negative group. When surgery or a non-operative approach was used, the relationship between CTC and OS was not statistically significant (P>0.05). The positive rate of CTC was associated with shorter OS in longer study time (P<0.05). When the study time was short, the relationship between CTC and OS was not statistically significant (P>0.05).

In the subgroup analysis of national factors, it was found that CTC positive was associated with shorter OS. By performing a subgroup analysis of CTC assays, it was found that by applying immunofluorescence assays, CTC positivity was associated with a shorter OS (**Table 2**).

**Table 2:** Subgroup analysis of the relationship between CTC and OS in patients with gastric cancer.

Subgroup analysis	Research number (n)	Sample size (n)	Model	*HR *(95% *CI*)	*P*	Heterogeneity	
						*I^2 ^* (%)	*P_h_*
Critical value of CTC positive							
≥2.8	7	445	Fixed effect model	1.76 (0.96˜3.21)	0.07	0	0.73
<2.8	7	608	Fixed effect model	2.54 (1.36˜4.74)	0.003	0	0.97
Country							
China	6	472	Fixed effect model	2.85 (1.32˜6.16)	0.008	0	0.99
Other	8	581	Fixed effect model	1.82 (1.08˜3.08)	0.03	0	0.76
Sample size							
≥75	5	596	Fixed effect model	3.09 (1.56˜6.09)	0.001	0	0.93
<75	9	457	Fixed effect model	1.61 (0.92˜2.83)	0.10	0	0.95
Mode of treatment							
Operation	5	423	Fixed effect model	2.31 (0.98˜5.45)	0.06	0	0.53
Non-operation	3	121	Fixed effect model	1.74 (0.82˜3.69)	0.15	0	0.42
Mixed therapy	6	509	Fixed effect model	2.30 (1.17˜4.52)	0.02	0	0.99
Research time							
≥2.5 year	5	575	Fixed effect model	2.88 (1.44˜5.74)	0.003	0	0.90
<2.5 year	8	451	Fixed effect model	1.62 (0.88˜2.96)	0.12	0	0.88
CTC detection method							
Immunofluorescence	12	875	Fixed effect model	2.06 (1.27˜3.34)	0.003	0	0.91
Molecular detection	2	178	Fixed effect model	2.27 (0.85˜6.07)	0.1	0	0.69

### Relationship between CTC and clinicopathological features of patients with gastric cancer

This study explored the relationship between CTC and clinicopathological features of gastric cancer patients from the aspects of age, sex, TNM stage, tumor differentiation, distant metastasis, Lauren classification, and CEA (**Table 3**). The heterogeneity of each study was small. The results of the meta-analysis showed that the CTC-positive patients had a higher TNM stage (*OR*=3.50, 95% *CI*=[2.21,5.54], *P<*0.00001), poorer tumor differentiation (*OR*=2.49, 95% *CI*=[1.54,4.03], *P*=0.0002), and earlier distant metastasis (*OR*=2.03, 95% *CI*=[1.36,3.04], *P*=0.0006); while the positive rate of CTC was not related to age, sex, Lauren classification and CEA (P>0.05).

**Table 3:** Relationship between CTC and clinicopathological features of patients with gastric cancer.

Pathological features	Research number (*n*)	Sample size (*n*)	Model	*OR *(95% *CI*)	*P*	Heterogeneity	
						*I^2^* (%)	*P_h_*
Age (high vs. low)	7	548	Fixed effect model	1.02 [0.67,1.55]	0.92	0	0.55
Gender (male vs. female)	9	741	Fixed effect model	1.20 [0.82,1.78]	0.35	0	1
TNM (III-IV vs. I-II)	7	684	Fixed effect model	3.50 [2.21,5.54]	<0.00001	49	0.07
Differentiation (poor vs. good)	6	517	Fixed effect model	2.49 [1.54,4.03]	0.0002	0	0.85
Distant metastasis (yes vs. no)	9	741	Fixed effect model	2.03 [1.36,3.04]	0.0006	17	0.29
Lauren (diffuse vs. no-diffuse)	4	317	Fixed effect model	1.53 [0.82,2.85]	0.18	45	0.14
CEA(high expression vs. low expression)	4	375	Fixed effect model	1.54 [0.89,2.66]	0.12	0	0.48

### Sensitivity analysis

**Figure 4:**
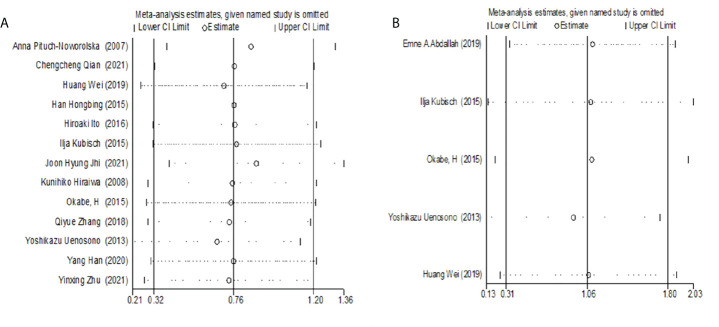
**(A)** Sensitivity Analysis of the relationship between CTC and OS. **(B)** Sensitivity analysis of the relationship between CTC and PFS.

Single studies were excluded one by one for sensitivity analysis. The results showed that the results of a meta-analysis analyzing the relationship between CTC and OS or PFS were stable (OS: *HR*= 0.67- 0.86; PFS: *HR*=0.93- 1.10) (**Figure 4**).

### Publication bias

**Figure 5:**
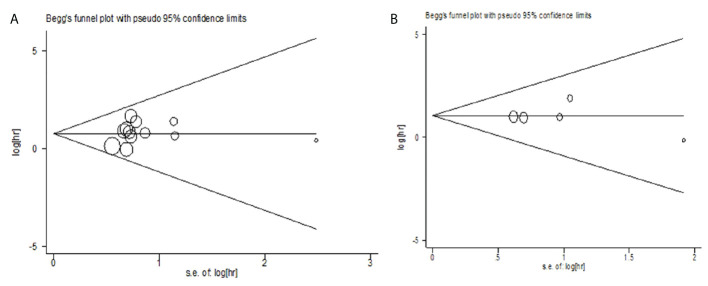
**(A)** The relationship between CTC and OS. **(B)** The relationship between CTC and PFS.

The publication bias of the relationship between CTC and OS was evaluated by the Begg test (*Z=*0.34, *P=*0.732) and Egger test (*t=*0.75, *P=*0.468). The publication bias of the relationship between CTC and PFS was analyzed by Begg test (*Z* =-0.24, *P* = 1.000) and Egger test (*t* = 0.33, *P* = 0.762). The results showed less possibility of publication bias in the included study (**Figure 5**).

## Discussion

As a new prognostic marker, CTC has the biological characteristics of the primary tumor and strong invasive ability (7). The release of CTCs from the tumor into the circulating blood occurs through the epithelial-mesenchymal transition (EMT) and non-EMT-mediated invasion (36). As a non-invasive and simple “fluid biopsy” technique, CTC detection is a simple procedure (37). Herein, we conducted a meta-analysis to evaluate the relationship between circulating tumor cells (CTC) and the prognosis of patients with gastric cancer. This study included the original studies from Chinese and English databases, including South Korea, Japan, China, and other Asian countries with a high incidence of gastric cancer, in order to improve the scientific and reliable conclusion of the relationship between CTC and the prognosis of patients with gastric cancer. Fourteen retrospective cohort studies with 1053 patients were included to explore the prognostic role of CTC in gastric cancer. The results showed that the positive rate of CTC was associated with shorter OS and PFS. CTC are tumor cells that detach from the primary or metastatic focus of the tumor and enter the blood (38). In recent years, CTC has been used as an important prognostic marker for many solid tumors, including lung cancer (39), breast cancer (40), prostate cancer (41), nasopharyngeal cancer (42), rectal cancer (43), and so on. CTC detection can effectively make up for the deficiency of imaging, serum markers, and tissue samples in the evaluation of the prognosis of patients with gastric cancer, providing qualitative, specific, and dynamic evaluation, and avoiding temporal and spatial heterogeneity of tumors. CTC can be directly detected through blood samples, which is helpful for clinicians to systematically and effectively evaluate the progression of tumors so as to provide scientific and reasonable treatment.

In this study, subgroup analysis indicated that critical value of CTC positive < 2.8, sample size ≥ 75, mixed therapy, long study time, and using immunofluorescence assay were associated with shorter OS in CTC-positive patients. However, the critical value of positive CTC ≥ 2.8, sample size < 75, simple surgical or non-operative treatment, short research time, and molecular detection method had no significant relationship with OS. This may be the reason why the small sample size and short research time could not reveal the real results, but it also shows that mixed treatment is the best choice for patients with gastric cancer.

After analyzing the subgroups of different countries, it was found that the positive rate of CTC was related to the shorter OS. There was no heterogeneity in the whole subgroup analysis (*I^2^* = 0%), ensuring the reliability of the research results. In addition, this study also explored the relationship between CTC and clinicopathological features of gastric cancer patients. CTC-positive patients had an advanced TNM stage, poorer tumor differentiation, and were more prone to distant metastasis than those with CTC-negative patients. These clinical parameters, which are closely related to the progression of malignant tumors, are correlated with CTC, which proves that the positive expression of CTC is an important index for evaluating the prognosis of patients with gastric cancer, which also indicates that the change of CTC in the process of tumor development may be the key factor causing tumor recurrence and metastasis, which is consistent with previous study (44).

This study has some limitations. First, only a few literature and retrospective cohort studies were included; also, there is a lack of data support for large samples of randomized controlled trials. At present, there is no unified positive standard of CTC in gastric cancer, which may lead to bias. In subgroup analysis, all heterogeneities could not be explored. Because only the baseline CTC count was collected in the included studies, the changes in CTC after an intervention such as surgery and chemotherapy were not analyzed, and it was impossible to evaluate the effect of treatment intervention on the prognosis of patients with gastric cancer. In their large sample size meta-analysis, Zou *et al.* (45) found that high CTC counts before and during chemotherapy were significantly correlated with poor OS, PFS, and disease control rates (DC) in patients with advanced gastric cancer. Moreover, Yue *et al.* (46) found that the dynamic changes in CTC and prognosis were also affected by the study of the relationship between gastrointestinal tumors and CTC.

## Conclusion

The existing evidence shows that CTC can be used as an effective index to evaluate the prognosis of gastric cancer. However, due to the research quantity and quality limitation, larger, high-quality studies are needed to further verify the above conclusions.

## Data availability statement

The original contributions presented in the study are included in the article/supplementary material. Further inquiries can be directed to the corresponding author.

## Author contributions

JY contributed to study design. ZL were responsible for literature search, data extraction and analysis and wrote the manuscript. MS, CJ and SH contributed to study selection. All authors contributed to the article and approved the submitted version.

## Funding

Natural Science Foundation of Gansu provincial (No. 21JR1RA017); Natural Science Foundation of Gansu provincial hospital (No. 21GSSYB-5).

## Conflict of interest

The authors declare that the research was conducted in the absence of any commercial or financial relationships that could be construed as a potential conflict of interest.

## Publisher's note

All claims expressed in this article are solely those of the authors and do not necessarily represent those of their affiliated organizations, or those of the publisher, the editors and the reviewers. Any product that may be evaluated in this article, or claim that may be made by its manufacturer, is not guaranteed or endorsed by the publisher.
